# Behavioral phenotypes of mice lacking purinergic P2X_4 _receptors in acute and chronic pain assays

**DOI:** 10.1186/1744-8069-5-28

**Published:** 2009-06-11

**Authors:** Makoto Tsuda, Kazuya Kuboyama, Tomoyuki Inoue, Kenichiro Nagata, Hidetoshi Tozaki-Saitoh, Kazuhide Inoue

**Affiliations:** 1 Department of Molecular and System Pharmacology, Graduate School of Pharmaceutical Sciences, Kyushu University, 3-1-1 Maidashi, Higashi-ku, Fukuoka 812-8582, Japan

## Abstract

A growing body of evidence indicates that P2X receptors (P2XRs), a family of ligand-gated cation channels activated by extracellular ATP, play an important role in pain signaling. In contrast to the role of the P2X_3_R subtype that has been extensively studied, the precise roles of others among the seven P2XR subtypes (P2X_1_R-P2X_7_R) remain to be determined because of a lack of sufficiently powerful tools to specifically block P2XR signaling *in vivo*. In the present study, we investigated the behavioral phenotypes of a line of mice in which the *p2rx4 *gene was disrupted in a series of acute and chronic pain assays. While *p2rx4*^-/- ^mice showed no major defects in pain responses evoked by acute noxious stimuli and local tissue damage or in motor function as compared with wild-type mice, these mice displayed reduced pain responses in two models of chronic pain (inflammatory and neuropathic pain). In a model of chronic inflammatory pain developed by intraplantar injection of complete Freund's adjuvant (CFA), *p2rx4*^-/- ^mice exhibited attenuations of pain hypersensitivity to innocuous mechanical stimuli (tactile allodynia) and also of the CFA-induced swelling of the hindpaw. A most striking phenotype was observed in a test of neuropathic pain: tactile allodynia caused by an injury to spinal nerve was markedly blunted in *p2rx4*^-/- ^mice. By contrast, pain hypersensitivity to a cold stimulus (cold allodynia) after the injury was comparable in wild-type and *p2rx4*^-/- ^mice. Together, these findings reveal a predominant contribution of P2X_4_R to nerve injury-induced tactile allodynia and, to the lesser extent, peripheral inflammation. Loss of P2X_4_R produced no defects in acute physiological pain or tissue damaged-induced pain, highlighting the possibility of a therapeutic benefit of blocking P2X_4_R in the treatment of chronic pain, especially tactile allodynia after nerve injury.

## Findings

The purinergic P2X receptors, of which seven subtypes (P2X_1_–P2X_7_) have been cloned, are a family of ligand-gated cation channels activated by extracellular ATP. They have important roles in regulating neuronal and glial functions in the nervous system under physiological and pathological conditions [1-3]. There has been much recent attention paid to their roles in generating and modulating pain signaling [1-3]. Various attempts to block P2XRs pharmacologically or to suppress their expression molecularly and genetically have demonstrated that these receptors make a major contribution to pain responses evoked by tissue damage, chronic peripheral inflammation and nerve injury [4-6]. While the role of P2X_3_R (and P2X_2+3_R) has been studied extensively, recent evidence from our and other studies indicates that the P2X_4_R subtype critically contributes to neuropathic pain, a highly debilitating pain condition that commonly occurs after nerve damage [7-13]. However, the precise role of P2X_4_R in neuropathic pain remains to be fully determined because, in stark contrast to P2X_3_R [14-16] and P2X_7_R [17,18], there is a lack of sufficiently powerful pharmacological and genetic tools to selectively block P2X_4_R signaling *in vivo*. Recently, several groups have independently developed lines of mice in which the *p2rx4 *gene is disrupted [19-21]. It is of particular importance to study the phenotypes of these mice to determine the *in vivo *functions of P2X_4_R in pain signaling [22]. In the present study, we sought to characterize behavioral phenotypes in a wide range of assays of acute and chronic pain, including neuropathic pain, using a line of P2X_4_R-deficient mice [19].

**Figure 1:**
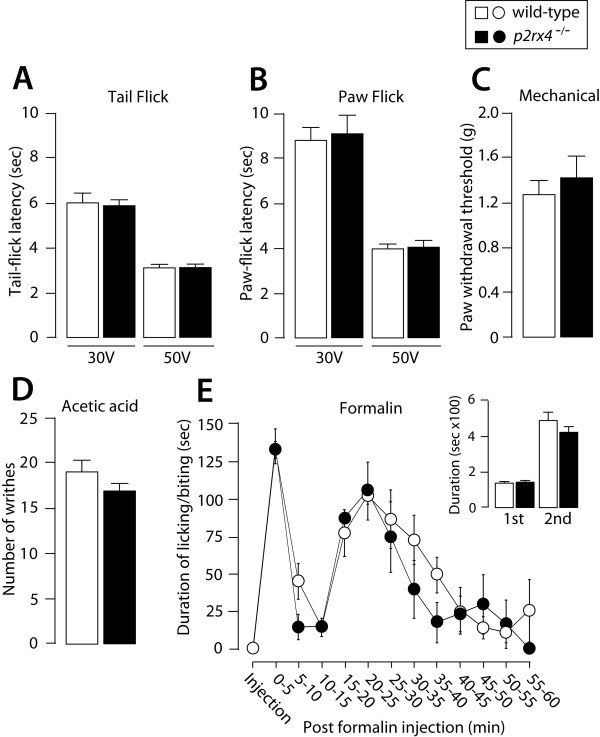
**Acute thermal and mechanical pain and chemical-induced pain in *p2rx4*^-/- ^mice**. (A) Tail-flick and (B) paw-flick tests. Values represent the latency (sec) for animals to flick their tail or paw away from the heat source (wild-type, n = 5; *p2rx4*^-/-^, n = 4). (C) Mechanical pain test. Values indicate the threshold (g) to elicit paw withdrawal behavior in response to mechanical stimuli (wild-type, n = 5; *p2rx4*^-/-^, n = 4). (D) Visceral pain in response to acetic acid (0.8%). Values represent the numbers of abdominal stretches (writhes) (wild-type, n = 6; *p2rx4*^-/-^, n = 5). (E) Formalin test. Mice were injected intraplantarly with formalin (5%, 20 μL). Values represent the duration (sec) of licking and biting responses for each 5-min interval (E), from 0 to 5 min (1st phase) and for 10–60 min (2nd phase) (inset) (wild-type, n = 8; *p2rx4*^-/-^, n = 6). All data are presented as means ± SEM.

To examine acute physiological pain responses, the withdrawal responses from a noxious range of heat and mechanical stimuli applied by von Frey filaments were measured in both wild-type and *p2rx4*^-/- ^mice. In tail- and paw-flick tests, the latencies for animals to flick their tails and hindpaws away from radiant heat at either 30 V or 50 V were not different between wild-type and *p2rx4*^-/- ^mice (Fig. 1A, B). In a test of mechanical pain, *p2rx4*^-/- ^mice were indistinguishable from wild-type mice in terms of their paw withdrawal thresholds (Fig. 1C). Abdominal writhing behavior in response to intraperitoneal injection with acetic acid, a model of chemical-induced visceral pain, was also comparable between the two lines of mice (Fig. 1D). To determine the role of P2X_4_R in tissue injury-induced acute and persistent pain, we assessed the pain response following the injection of formalin into the hindpaw. In wild-type mice, injection of formalin elicited biphasic biting and licking behaviors: the first phase started immediately after the injection and lasted for 5 min, and the second phase lasted for 60 min (Fig. 1E). Neither the pattern nor the magnitude of biphasic behaviors was altered in *p2rx4*^-/- ^mice (Fig. 1E). The amount of swelling of the formalin-injected hindpaw, as indicated by an increase in the weight of the hindpaw 60 min after the injection, was not significantly different between the two lines of mice (contralateral hindpaw: wild-type 0.137 ± 0.008 g, *p2rx4*^-/- ^0.130 ± 0.004 g; ipsilateral: wild-type 0.173 ± 0.005 g, *p2rx4*^-/- ^0.181 ± 0.005 g). These results indicate that pain signaling elicited by acute noxious stimuli and tissue damage is intact in *p2rx4*^-/- ^mice.

**Figure 2:**
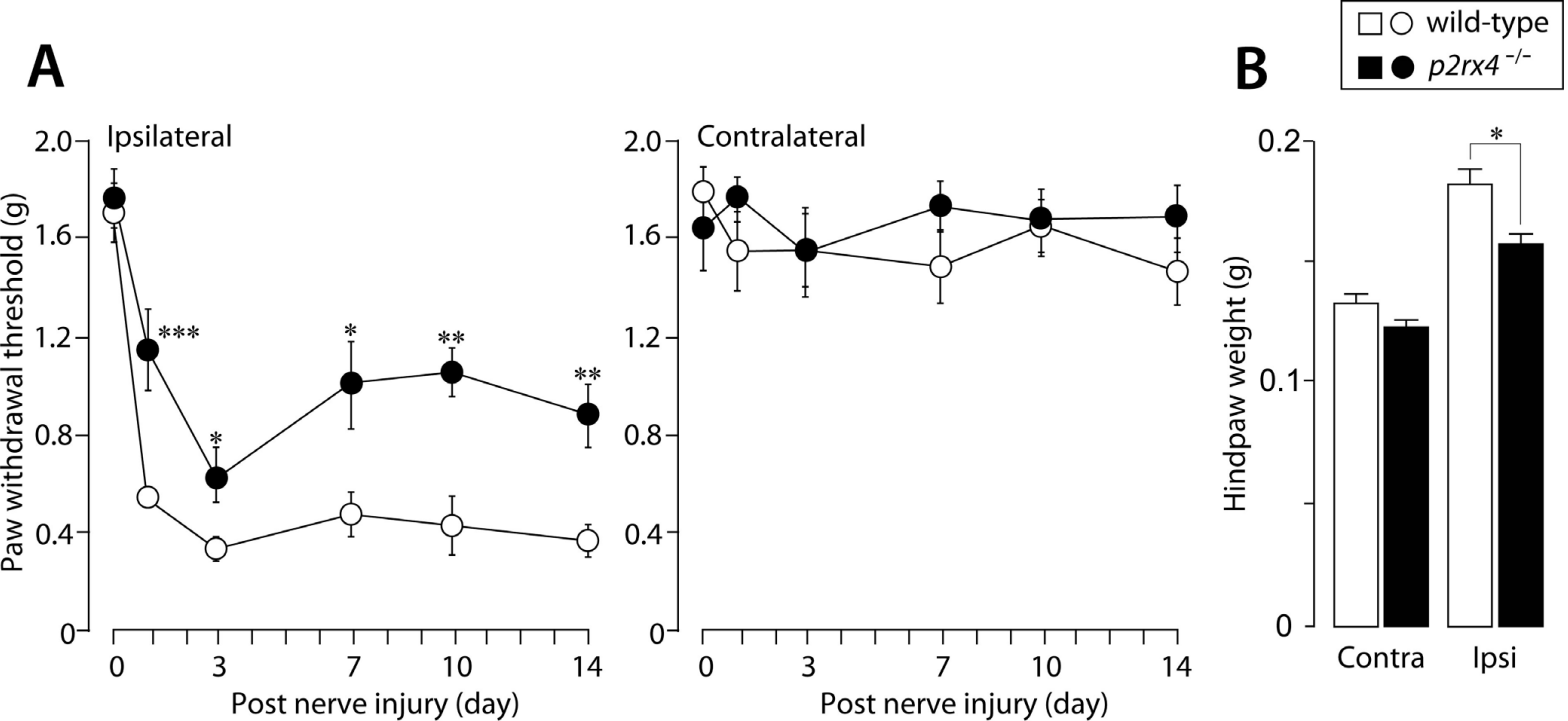
**Reduction in the amount of peripheral inflammation-induced pain in *p2rx4*^-/- ^mice**. (A) Paw withdrawal thresholds (left panel, ipsilateral hindpaw; right panel, contralateral hindpaw) of wild-type (n = 8) and *p2rx4*^-/- ^mice (n = 8) before (0) and 1, 3, 7, 10 and 14 days after intraplantar injection of CFA (0.01 mg/20 μL). *p < 0.05, **p < 0.01, ***p < 0.001 vs. wild-type mice. (B) Change in the weight (g) of the ipsilateral and contralateral hindpa0w 14 days after the injection of CFA (wild-type, n = 6; *p2rx4*^-/-^, n = 6). *p < 0.05 vs. wild-type mice. All data are presented as means ± SEM.

Next, to investigate the role of P2X_4_R in pain hypersensitivity under chronic pain conditions, we employed two distinct models of chronic pain, namely inflammatory and neuropathic pain. Wild-type mice with peripheral inflammation induced by intraplantar injection of complete Freund's adjuvant (CFA), used as a model of inflammatory pain, displayed decreased paw withdrawal thresholds in their ipsilateral hindpaws (Fig. 2A). By contrast, the peripheral inflammation-induced decrease in paw withdrawal threshold was significantly smaller in *p2rx4*^-/- ^mice than wild-type mice. The paw withdrawal thresholds for the contralateral hindpaws in both genotypes of mice did not change (Fig. 2A). In wild-type mice, a marked swelling of the ipsilateral hindpaw was observed, and the weight of the hindpaw was increased on day 14 (Fig. 2B). In *p2rx4*^-/- ^mice, however, the CFA-induced increase in the weight of the ipsilateral hindpaw was significantly suppressed (*p *< 0.05, Fig. 2B).

**Figure 3:**
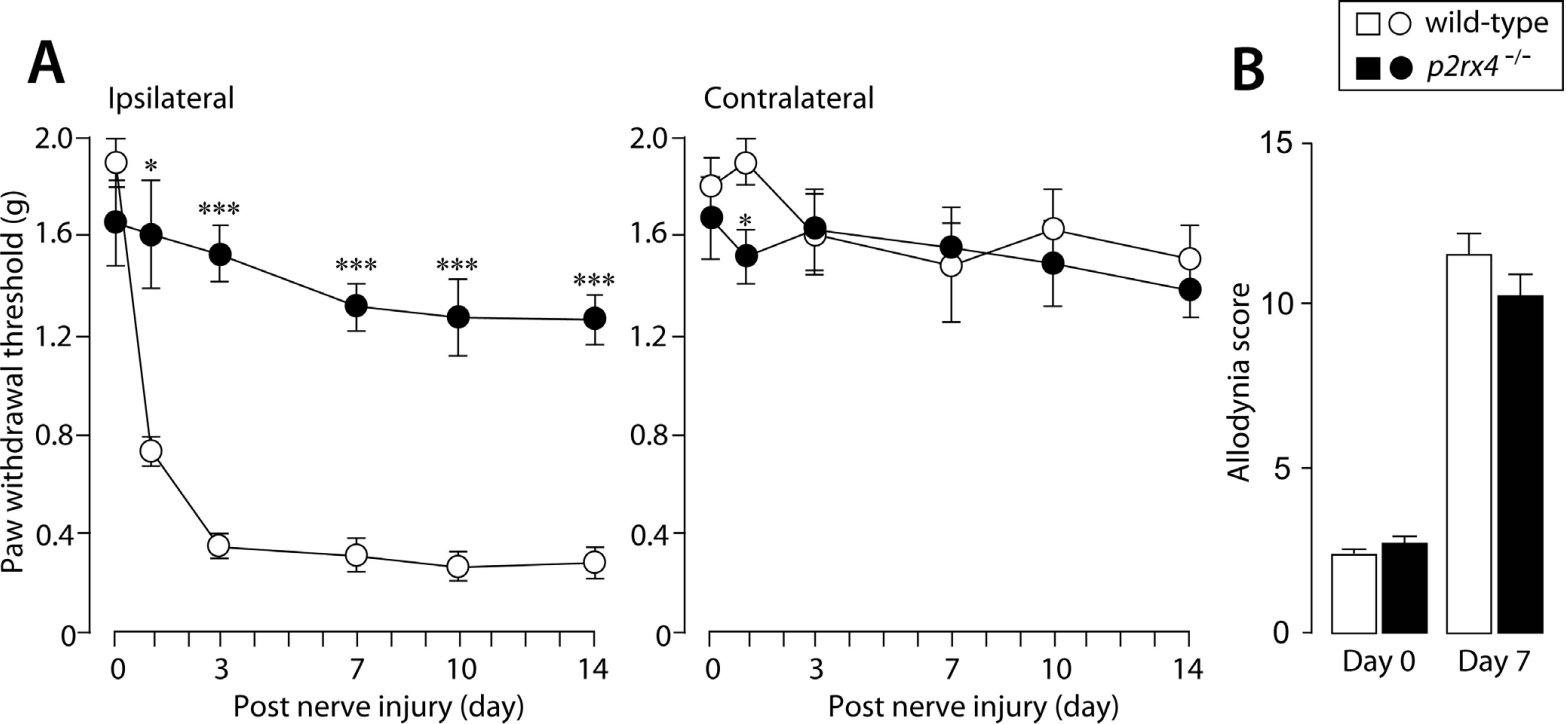
**Nerve injury-induced tactile allodynia is markedly blunted in *p2rx4*^-/- ^mice**. Paw withdrawal thresholds (left panel, ipsilateral hindpaw; right panel, contralateral hindpaw) of wild-type (n = 5) and *p2rx4*^-/- ^mice (n = 6) before (0) and 1, 3, 7, 10 and 14 days after spinal nerve injury. (B) Cold allodynia evoked by applying acetone to the plantar surface of the ipsilateral hindpaw 7 days after nerve injury (wild-type, n = 5; *p2rx4*^-/-^, n = 5). *p < 0.05, ***p < 0.001 vs. wild-type mice. All data are presented as means ± SEM.

To determine the role of P2X_4_R in tactile allodynia under neuropathic pain conditions, we injured the forth lumbar spinal nerves of wild-type and *p2rx4*^-/- ^mice; this approach has been used to generate an animal model of neuropathic pain. While wild-type mice showed a decrease in paw withdrawal threshold after nerve injury (Fig. 3A), this effect was markedly blunted in *p2rx4*^-/- ^mice. The attenuated tactile allodynia was observed until the last time point tested (day 1, *p *< 0.05; day 3, 7, 10 and 14, *p *< 0.001; Fig. 3A). The loss of P2X_4_R did not change the paw withdrawal threshold of the contralateral hindpaw after nerve injury (Fig. 3A) as seen in wild-type mice (except on day 1, *p *< 0.05 vs. wild-type, Fig. 3A). A reduction in pain behaviors is occasionally misinterpreted as a result of non-specific motor dysfunction, but the rotarod performance test demonstrated no significant difference in the time on the rotarod between the two genotypes (data not shown). We further tested whether P2X_4_R deficiency also suppresses nerve injury-induced hypersensitivity to cold stimulation, a phenomenon known as cold allodynia. In striking contrast to the lack of nerve injury-induced tactile allodynia in *p2rx4*^-/- ^mice (Fig. 3A), both wild-type and *p2rx4*^-/- ^mice showed markedly enhanced responsiveness to a cold stimulus evoked by applying acetone to the ipsilateral hindpaw after nerve injury (Fig. 3B). There was no difference in the behavioral response to acetone in the contralateral hindpaw between the two lines of mice (wild-type, 3.2 ± 0.3; *p2rx4*^-/-^, 3.2 ± 0.4). These results indicate that P2X_4_R deficiency leads to a striking reduction in the tactile allodynia caused by nerve injury and, to the lesser extent, peripheral inflammation, without any defects in motor function.

By using P2X_4_R-deficient mice, we revealed that P2X_4_R is not required for all forms of pain responses, but rather is crucial for specialized pain states, namely chronic inflammatory and, in particular, neuropathic pain. Our present results showing the striking reduction of tactile allodynia after an injury to spinal nerves in *p2rx4*^-/- ^mice together with our previous findings in rats [7] and recent findings in a line of *p2rx4*^-/- ^mice after an injury to the sciatic nerve [22] provide compelling evidence for an essential role of P2X_4_R in nerve injury-induced tactile allodynia. To elucidate the mechanisms underlying the blunted neuropathic allodynia in *p2rx4*^-/- ^mice requires further investigations, but it may involve a lack of microglial P2X_4_Rs in the spinal cord, expression of which is markedly upregulated after nerve injury [7,22]. Stimulation of P2X_4_R in microglia induces release of brain-derived neurotrophic factor [9,22,23], a factor that is crucial for producing aberrant excitability of dorsal horn neurons [9,24]. Therefore, the attenuated neuropathic allodynia in *p2rx4*^-/- ^mice may be associated with a reduction in the amount of pathologically altered neurotransmission in dorsal horn neurons caused by microglia-derived BDNF [22].

An interesting finding in the present study was that nerve injury-induced cold allodynia was retained in *p2rx4*^-/- ^mice, indicating that P2X_4_R may have distinct roles in mechanical and cold hypersensitivities after nerve injury. In a recent study it was shown that inhibition of Src-family kinases (SFKs), whose activity is enhanced in spinal microglia after nerve injury, results in suppression of tactile allodynia without affecting cold allodynia [25]. Our previous study has implicated Lyn (microglial SFK) as a critical kinase causing P2X_4_R upregulation [13]. It is thus conceivable that P2X_4_R-dependent microglial signaling in the dorsal horn may participate predominantly in allodynia evoked by mechanical rather than cold stimulation after peripheral nerve injury.

Our present study showed a reduction in the level of CFA-induced inflammatory pain in *p2rx4*^-/- ^mice. In contrast to the neuropathic pain model, neither the upregulation of P2X_4_R expression [7] nor activation of Lyn tyrosine kinase [13] has been demonstrated in spinal microglia following peripheral inflammation caused by CFA. The mechanisms underlying the reduction in the amount of inflammatory pain remain unknown, but the phenotype may be related to a reduction in hindpaw inflammation in *p2rx4*^-/- ^mice. This notion is supported by evidence of expression of functional P2X_4_Rs in peripheral inflammatory cells, including macrophages [21,26].

In conclusion, by employing a series of acute and chronic pain tests using mice lacking P2X_4_R, we demonstrated that the loss of P2X_4_R leads to a marked reduction in the degree of tactile allodynia caused by nerve injury and, to a lesser extent, in peripheral inflammation, without any defects in motor function. By contrasting the roles of P2X_4_R in distinct types of nerve injury-induced pain hypersensitivities, namely tactile and cold allodynia, the present study demonstrates a predominant contribution of P2X_4_R to tactile allodynia rather than cold allodynia. The fact that acute physiological pain and tissue damage-induced pain were normal in *p2rx4*^-/- ^mice highlights the possibility of a therapeutic benefit of blocking P2X_4_R in the treatment of chronic pain, especially tactile allodynia after nerve injury.

## Methods

### Animals

All experimental procedures were performed under the guidelines of Kyushu University. Male mice lacking P2X_4_R (*p2rx4*^-/-^) that were backcrossed to C57BL/6J (Clea Japan) for more than 10 generations were kindly provided by Prof. Joji Ando (The University of Tokyo) [19], and we used C57BL/6J as the corresponding control mice. All mice were used at age of 9~11-week-old (at the start of each experiment). Mice were housed in groups of 2~3 per cage at a temperature of 22 ± 1°C with a 12-h light-dark cycle (light on 8:30 to 20:30), and fed food and water *ad libitum*.

### Behavioral assays for acute and chronic pain

Noxious heat-evoked tail and hindpaw withdrawal responses were detected by the application of radiant heat (Ugo Basile, Italy) to the tail and the plantar surface of hindpaw, respectively [27,28]. The intensity of the heat stimulus was adjusted to 30 or 50 V, and the latency of the paw withdrawal response (sec) was measured. The sensitivity to mechanical stimulus was assessed using von Frey filaments (0.02~2.0 g, Stoelting, Wood Dale, Illinois, USA), and the mechanical stimulus producing the 50% paw withdrawal threshold was determined using the up-down method [29,30]. In the tests of formalin-induced pain, mice were injected intraplantarly with formalin (5%, 20 μL), and then the duration of the licking and biting responses to the injected hindpaw was recorded at 5 min intervals for 60 min after the injection (formalin pain) [31,32]. For the measurement of hindpaw swelling by formalin, the weights of the hind feet amputated at the ankle were measured 60 min and 14 days after the injection of formalin and CFA, respectively [32]. In the chemical visceral pain test, mice were injected intraperitoneally with acetic acid (0.8%), and the number of abdominal writhes was counted for 5 min starting from 5 min after the injection [32]. Motor coordination was assessed using the rotarod performance test [31]. For the inflammatory pain model, CFA (0.01 mg/20 μL) was injected into the plantar surface of the left hindpaw [13]. For the neuropathic pain model, the left L4 spinal nerve of mice was transected under isoflurane (2%) anesthesia [33-35]. To assess the tactile allodynia, mice were placed individually in an opaque plastic cylinder which was placed on a wire mesh and habituated for 1 hr to allow acclimatization to the new environment. After that, calibrated von Frey filaments (0.02–2.0 g, Stoelting) were applied to the plantar surface of the hindpaw from below the mesh floor, and the 50% paw withdrawal threshold was determined. To assess cold allodynia [36], a drop (50 μL) of acetone was placed against the centre of the plantar surface of the hindpaw and a stopwatch was started. The mouse's response was monitored in the first 20 sec after acetone application. If the mouse did not withdraw, flick or stamp its hindpaw within this 20-sec period then no response was recorded for that trial (0). However, if within this 20 sec period the animal responded to the cooling effect of the acetone, then the animal's response was assessed for an additional 20 sec (a total of 40 sec from initial application). Responses to acetone were graded according to the following four-point scale: 0, no response; 1, quick withdrawal, flick or stamp of the paw; 2, prolonged withdrawal or repeated flicking (more than 2 times) of the paw; 3, repeated flicking of the paw with licking directed at the plantar surface of the hindpaw. Acetone was applied alternately five times to each hindpaw and the responses were scored categorically. Cumulative scores were then generated for each mouse.

### Statistical Analysis

The statistical analyses of the results were evaluated by using the Student’s *t* test or the Mann-Whitney *U *test.

## Competing interests

The authors declare that they have no competing interests.

## Authors' contributions

MT designed, performed and supervised the experiments, analyzed the data, and wrote the manuscript; KK and TI performed the experiments; KN, HST. analyzed the data; KI coordinated the project, helped to interpret the data, and edited the manuscript. All authors have read and approved the final manuscript.
